# Usability Testing of an Internet-Based Responsive Parenting Program for Caregivers of Young Survivors of Childhood Cancer Living in Rural and Appalachian Communities: Mixed Methods Study

**DOI:** 10.2196/70055

**Published:** 2025-08-05

**Authors:** Emily L Moscato, Eva Darow, Jessica Quach, Adrien M Winning, Matthew Schmidt, Shari L Wade, Cynthia A Gerhardt, Emre Sezgin

**Affiliations:** 1 The Center for Biobehavioral Health, The Research Institute, Nationwide Children's Hospital, 431 S 18th St, Columbus, OH, 43205, United States, 614-722-4724; 2 Department of Pediatrics, College of Medicine, The Ohio State University, Columbus, OH, United States; 3 Department of Workforce Education and Instructional Technology, Mary Frances Early College of Education, University of Georgia, Athens, GA, United States; 4 Department of Clinical and Administrative Pharmacy, College of Pharmacy, University of Georgia, Athens, GA, United States; 5 Division of Rehabilitation Medicine, Cincinnati Children's Hospital Medical Center, Cincinnati, OH, United States; 6 Department of Pediatrics, College of Medicine, University of Cincinnati, Cincinnati, OH, United States

**Keywords:** pediatrics, oncology, parenting, rural, Appalachian

## Abstract

**Background:**

Young survivors of childhood cancer diagnosed and treated before the age of 7 years are at heightened risk of developmental difficulties. As a result, their caregivers may experience stress as they navigate various educational and health care systems while advocating for their child’s needs. To our knowledge, there are no tailored early interventions or support programs to address the unique and multifaceted needs of young survivors of childhood cancer and their caregivers. Digital platforms can provide anticipatory guidance and developmental resources, which may be especially impactful for families at increased risk for health and educational disparities, particularly in underserved rural and Appalachian areas.

**Objective:**

This mixed methods study describes the usability testing of an internet-based responsive parenting program for caregivers of young survivors of childhood cancer, called the Preparing for Life and Academics for Young Survivors (PLAY) program.

**Methods:**

Twelve caregivers of young survivors of childhood cancer (9 biological parents, 2 grandfathers, and 1 legal guardian; 33% rural, 33% Appalachian, and 83% White) participated in an online usability session with a task-based, think-aloud method. This session was followed by completion of the System Usability Scale (SUS), responses to reaction cards to describe their experiences with the platform, and a semistructured interview. Two trained coders used rapid analysis and consensus methods to identify themes.

**Results:**

Overall, caregivers found the platform easy to use (92% SUS >68; mean 82.2, SD 14.45). Most caregivers provided positive feedback in response to reaction cards (eg, useful, appealing, and valuable) to describe the platform. Qualitative findings highlighted that most caregivers perceived the platform as “easy to use,*”* and several participants described it as a helpful educational tool. Participants noted that potential platform improvements should include accessibility features (eg, closed captioning for videos), more tailored content, and usability on mobile devices.

**Conclusions:**

With further improvements, digital platforms such as PLAY may be a promising avenue to bridge access to care for high-need groups and ultimately improve child neurodevelopmental outcomes.

## Introduction

Survival rates for most types of childhood cancers have fortunately risen to over 80%, resulting in a growing cohort of nearly half a million childhood cancer survivors in the United States [1]. However, life-saving cancer treatments, such as chemotherapy and radiation, can be detrimental to a child’s development and lead to a host of chronic health needs for up to two-thirds of survivors, termed late effects [1]. Late effects from cancer treatment can vary and include impacts on physical, cognitive, social, and emotional development that may impair long-term academic functioning and quality of life [1-4]. Cancer treatment is particularly disruptive to early development, with young survivors of childhood cancer who are diagnosed and treated before the age of 7 years at heightened risk for chronic late effects [5-7].

The emotional toll of cancer extends to caregivers, who are faced with the daily realities of caring for a young child with cancer [8]. Even after cancer, caregivers may need to manage chronic medical needs and heightened anxiety about recurrence, with up to 40% of caregivers having clinically significant levels of post-traumatic stress symptoms [9]. Caregivers have reported a high need for information about their child’s medical condition and future late effects [10]. Parenting stress has also been linked with poorer developmental outcomes for young survivors of childhood cancer [11], further exacerbating the detrimental impact of cancer treatment. In other neurologically vulnerable populations, such as those with traumatic brain injury (TBI) and preterm birth, responsive parenting programs that teach parents how to increase warmth and cognitive stimulation and take care of themselves may lead to improvements in child developmental outcomes [12,13], suggesting potential intervention opportunities for young survivors of childhood cancer.

Still, young survivors of childhood cancer and their families living in underserved rural or Appalachian areas have barriers to survivorship care. Appalachia is a 250,000-square-mile region extending from New York to Mississippi and includes over 25.7 million residents [14]. This region has been plagued by economic distress, often resulting in residents with limited access to specialty health care due to financial barriers and distance [15]. Access to mental health care is further limited by stigma and misperceptions of the potential benefits of these services [16,17]. Although research is scarce in young survivors of childhood cancer, older CCS in rural regions have demonstrated deficits in social competence as compared to their nonrural counterparts [18,19]. Thus, to improve health equity, it is essential to identify accessible, evidence-based early interventions and psychosocial supports for young survivors of childhood cancer and their caregivers to mitigate disparities in outcomes.

Digital health interventions may be a potential solution to increase access to specialized care for underserved populations [20], such as rural and Appalachian young survivors of childhood cancer and their caregivers. Recent systematic reviews have identified over 30 studies (N=32) focused on digital health interventions for families of children with special health care needs [21]. A few of these studies identified additional barriers to care for rural pediatric populations, including poor internet connectivity, leading to poor video quality and interruptions to care [21,22]. Thus, to facilitate future implementation and uptake, and to promote health equity, user-centered design methods that begin at the earliest stages of intervention development are needed [23]. The Consolidated Framework for Implementation Research (CFIR) has identified innovation design, or the extent to which an intervention is accessible and easy to use, as a key component of uptake and implementation [24]. Furthermore, interventions that have included user-centered design have also demonstrated greater effectiveness [25,26].

**Figure 1.**
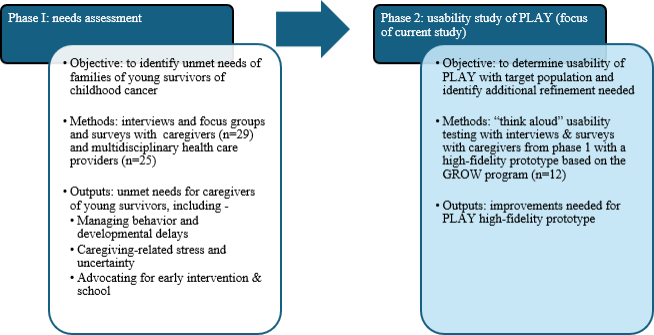
Overview of the development of the Preparing for Life and Academics for Young Survivors program, including the previous phase of research (phase 1 [Darow et al, report in preparation; Kaufman et al, report under review; and Moscato et al; report in preparation] and phase 2 [focus of this paper]). GROW: Gaining Real Life Skills Over the Web program; PLAY: Preparing for Life and Academics for Young Survivors.

To our knowledge, there are no tailored early intervention or support programs to address the unique and multifaceted needs of young survivors of childhood cancer and their caregivers, especially for those living in underserved rural or Appalachian areas. Therefore, we sought to develop a responsive parenting program for this population. Figure 1 provides an overview of this work. The findings from the needs assessment (phase 1; Darow et al, report in preparation; Kaufman et al, report under review; and Moscato et al; report in preparation) suggested several unmet psychosocial needs for young survivors of childhood cancer (eg, behavioral dysregulation, developmental delays, and impaired quality of life), as well as their caregivers (eg, caregiving-related stress, managing uncertainty, and advocacy related to early intervention services and school). Given that the unmet needs endorsed by caregivers of young survivors of childhood cancer were similar to those identified by caregivers of other neurologically vulnerable populations, such as individuals with TBI, we opted to tailor a previously developed internet-based intervention called the Gaining Real Life Skills Over the Web (GROW) program. The GROW program was designed for caregivers of young children after TBI (ages 0‐4 years) [27] and targeted responsive parenting and caregiver distress through psychoeducation and individualized coaching [28].

Following the needs assessment, our team tailored the wording of the content in GROW to fit the population-specific needs of young survivors of childhood cancer (eg, changing “brain injury” to “cancer experience”) in the adapted intervention, now called the Preparing for Life and Academics for Young Survivors (PLAY) program. We also opted to focus on young survivors of childhood cancer aged 3‐6 years for the PLAY program, given that state-based early intervention programs focus on 0‐3 years, and caregivers in phase 1 identified an unmet need related to the transition to preschool and kindergarten. The focus of this study (Figure 1, phase 2) is a preimplementation, mixed methods usability testing study [24] involving interviews and standardized measures with 12 caregivers of young survivors of childhood cancer using a high-fidelity prototype of the PLAY program. The purpose of this phase of the study is to inform the refinement of PLAY based on usability metrics, including both standardized measures (ie, the System Usability Scale [SUS] and acceptable usability indicated by scores >68) [29,30] and qualitative feedback from caregivers. The ultimate goal of this program of research is to develop an accessible program that will improve parenting stress and child outcomes for this vulnerable group of young survivors of childhood cancer, especially those in underserved rural and Appalachian areas.

## Methods

### Overview

We used a mixed methods research design with a sample of caregivers (n=12) to examine the usability of the PLAY high-fidelity prototype. A high-fidelity prototype is a simplified version representing the skeletal framework of a website or application’s interface [31]. Specifically, we used an expedited iterative design process to advance the study timeline. Rapid usability analysis has been used to speed the interpretation of user feedback and maximize identified codes and themes via reviewing conversations (ie, audio and video recording) instead of transcriptions [32-34]. This approach is known for its efficiency in distilling user feedback, accelerating the identification of codes and themes by analyzing audio and video recordings directly, thereby bypassing the labor-intensive transcription process. This method ensures swift progression from user feedback to design iteration, aligning with user-centered design principles [23]. By engaging a diverse cross-section of caregivers, including a significant representation from underserved rural or Appalachian regions (over 30%), we aimed to rapidly pinpoint and implement essential modifications to tailor the platform to the needs of these communities.

### PLAY High-Fidelity Prototype

A high-fidelity prototype of the PLAY website, based on GROW (Figure 1), was developed and shared with caregivers in this study to (1) determine usability among caregivers of young survivors of childhood cancer and (2) identify recommendations for tailoring the program to their unique needs. The PLAY website was based on the GROW website, a fully developed digital health intervention program originally designed for caregivers of young children ages 0‐4 years following TBI, to improve responsive parenting skills and cognitive stimulation [27,28]. Participants were instructed to review the content, as generally about having a child with a chronic medical condition. The GROW intervention involves internet-based learning modules (ie, GROW platform), as well as live one-on-one coaching via videoconferencing and weekly homework. GROW was based on a previously developed and tested responsive parenting program called PLAY and Learning Strategies (PALS) [35] and informed by caregivers and experts in special education and TBI. All 3 programs (ie, PALS, GROW, and PLAY) are rooted in attachment theory [36] and sociocultural models of child development [37]. The core treatment target is responsive parenting, which involves increasing caregiver warmth and contingent reactions to their child’s cues, while also providing verbal stimulation that enriches development.

The PLAY website, such as GROW, has six learning modules: (1) interactions, (2) language development, (3) attention/play, (4) parent self-care, (5) relationships, and (6) working with schools. Each module includes brief text content on the topic area, videos highlighting examples of caregivers interacting with their children and practicing the skills, and exercises to apply learned information, such as matching and quizzes (refer to previous publications) [27]. The interface was built using the popular content management platform WordPress (Automattic, Inc), augmented with the LearnDash plugin to provide learning management system functionality. This system is compatible with PC, mobile (Android and iOS), and tablet browsing due to an inbuilt responsive design.

### Procedure

Purposive sampling was used to identify potentially eligible caregivers of young survivors of childhood cancer from the cancer registry of a Midwest pediatric academic medical center. Inclusion criteria for eligible participants included caregivers of a child who (1) was diagnosed with cancer before the age of 7 years, (2) was between the ages of 3‐12 years at the time of study participation, (3) did not have a history of a neurodevelopmental disorder prior to their cancer diagnosis (eg, neurofibromatosis, tuberous sclerosis, Down syndrome, and autism), (4) had completed treatment for cancer at least 6 months prior or was on maintenance oral chemotherapy only, without evidence of recurrent disease or current hospitalization. Caregivers also were required to be (1) the child’s legal guardians, (2) living with the identified child at least 50% of the time, (3) fluent in English, and (4) residing in the state of the institution or surrounding states (ie, Ohio, Kentucky, Pennsylvania, or West Virginia). Note that families at different time points post treatment were recruited to provide a range of perspectives, and that caregivers of children currently aged 7‐12 years were asked to retrospectively consider unmet needs and the use of the PLAY program during the planned target age range of 3‐6 years.

Stratified purposive sampling was conducted by using random sampling within subgroups to ensure representation by diagnosis type (ie, brain tumor, leukemia/lymphoma, and solid tumor), geographic location (ie, rural, nonrural, Appalachian, and non-Appalachian as defined by the Rural Urban Commuting Codes [38] and Appalachian Regional Commission [14]), and age of the young survivors of childhood cancer (ie, either 3‐6 or 7‐12 years; to include those at various time points postcancer diagnosis/treatment). We also intentionally oversampled individuals from racial and ethnic minority groups to increase representation in our sample and reflect the institutional cancer registry (ie, 9% Black and 6% Latinx).

Caregivers were invited to participate with a mailed letter from their oncologist or neurosurgeon, which was followed by attempts to contact the caregiver by phone. Caregivers could opt out of future contact with the research team. In phase 1 of this study, caregivers were initially invited to participate in a mixed methods assessment of their unmet psychosocial and educational needs involving internet-based REDCap (Research Electronic Data Capture; Vanderbilt University) surveys and an interview conducted by phone or secure videoconferencing. Caregivers could also elect to participate in a monthly Community Advisory Board (CAB) and future research. Research staff discussed the CAB and usability testing with potentially interested members during their scheduled phone/videoconference interview until the desired sample size for planned usability testing was acquired (n of at least 10) [39] and saturation in rapid data analysis was attained.

Usability testing visits were scheduled at the convenience of caregivers on a secure videoconferencing platform with screen sharing and recording capabilities. All visits were recorded and transcribed automatically by the secure videoconferencing platform for cross-referencing during the coding process. During the usability testing visit, a trained research staff member who conducted phase 1 with the caregiver provided brief context on the platform (ie, currently designed for families of young children following TBI). Next, caregivers were provided with the link to the platform and were instructed to engage in unstructured navigation of the platform on their own for 5 minutes while sharing their screen (ie, hereafter “unguided use”). Caregivers were encouraged to “think aloud” about any of their feedback. Pages accessed and verbal comments were noted for analysis.

**Figure 2.**
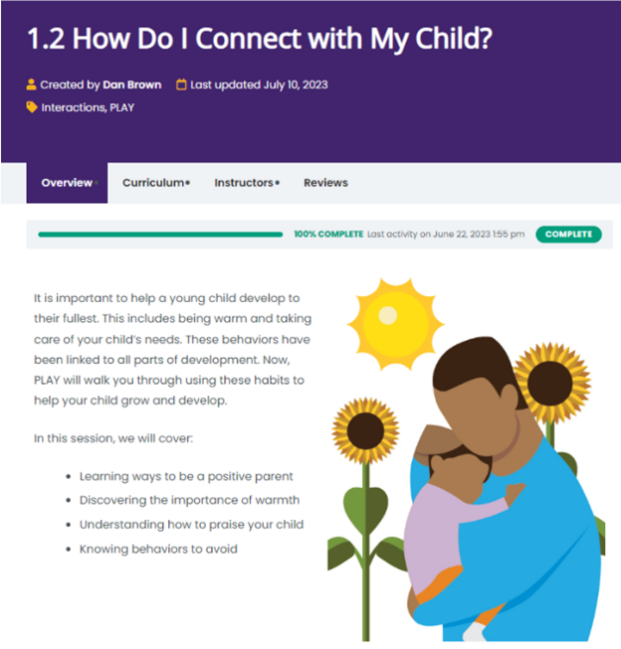
Sample module web page from the high-fidelity prototype of the responsive parenting platform for usability testing.

Following this, caregivers were instructed to perform a series of structured, task-based scenarios involving one specific module of the platform (refer to Figure 2; minimodule: “How Do I Connect with My Child?” and File S1 in Multimedia Appendix 1) for standardization across caregivers. This minimodule introduced the key concept of warmth, including how to use specific, labeled praises and the skill of diaphragmatic breathing for caregiver stress management. On each page of this module, research staff members provided standardized prompts (eg, “start the module,” “go to the next page,” “watch the video,” and “complete the exercise”) and encouraged caregivers to continue to “think aloud.” Total time to complete the scenario, navigation challenges, and verbal comments were noted for analysis. Following the completion of the scenario, caregivers completed brief online surveys (refer to the “Measures” section below) to indicate the device used (ie, mobile, tablet, or PC) and to provide feedback about the platform. Caregivers also engaged in a semistructured interview based on the technology acceptance model [40] about their overall impressions of the platform, other potential components of the intervention (ie, coaching by videoconferencing and text messaging), and suggestions for improvement. A copy of the semistructured interview guide is included in File S2 in Multimedia Appendix 1.

### Measures

#### Background Characteristics

In phase 1 of the study, caregivers completed a brief demographic survey including their relationship status, family income, employment status, race, ethnicity, and self-identification as Appalachian/non-Appalachian. The institutional cancer registry was also used to obtain information about the cancer diagnosis of young survivors of childhood cancer and to code for geographic region based on the family’s current address.

#### Device Usage

Caregivers completed a brief survey on the type of device used to access the platform (ie, smartphone, laptop/desktop computer, iPad/tablet, and other), the type of device they believed would be most useful for the platform (repeated options), and the selection of all devices available in their household.

#### System Usability Scale

Caregivers completed the 10-item SUS [30] to quantitatively assess usability. The SUS is a widely used, validated, and reliable measure of the acceptability and comprehensibility of the online program, ranging from 1 to 100, with higher scores indicating higher usability. A score more than 68 is considered acceptable/above average usability [29,41].

#### Reaction Cards

Caregivers selected words from an abbreviated list of 55 adjectives based on the Microsoft reaction cards [42] to describe their feelings and reactions toward the platform, providing qualitative insights into desirability and aesthetic design.

### Data Analysis Plan

Descriptive statistics (means and SDs) and frequency counts were calculated for demographic and medical variables, device usage, SUS scores, reaction cards, and documented data from the sessions (ie, pages accessed and length of time for scenario). For the qualitative data, a rapid analysis approach [32-34] was implemented with 2 trained research staff members, who served as interviewers and coders (EM, a postdoctoral fellow and licensed clinical psychologist, and ED, a clinical research coordinator). They attended usability testing sessions live and took detailed field notes. Recordings and field notes were reviewed a second time, with general codes extracted into summary templates organized by interview question in Microsoft Excel spreadsheets. Double coding was conducted for 25% of the interviews, with consensus reached prior to independent coding for the remainder of the data. A second consensus meeting was held to identify themes endorsed by a majority of participants across interviews using a matrix. Member checking was conducted with Caregiver Advisory Board members, who confirmed the findings and coding themes.

### Ethical Considerations

This study was reviewed and approved by the Nationwide Children’s Hospital’s institutional review board (00002939). As a minimal risk study, participants verbally agreed to participate after reviewing a written information sheet detailing risks and potential benefits. Participants also had an opportunity to ask questions of the study staff.

## Results

### Participants

Of 524 families in the cancer registry, 137 families were randomly selected as part of the stratification process in the “Procedure” section and were attempted to be contacted. Of those, 84 caregivers were reached by phone, and 32 caregivers agreed to participate in phase 1. A total of 25 agreed to be recontacted to participate in the CAB and other future studies, and the first 12 caregivers were sequentially invited for usability testing. There were no significant differences in demographic characteristics of the caregiver or child with cancer (ie, geographic region, sex, income, race, ethnicity, and diagnostic type) between the subsample of 12 participants who completed usability testing and the 32 participants from phase 1 (Fisher exact tests; *P*>.05), except that caregivers who completed usability testing were slightly older (mean 42.67 and SD 6.5 years) than those who did not participate in the usability phase (mean 36.8 and SD 6.8 years; *t*_30_=–2.41, *P*=.02).

A total of 12 usability sessions were conducted by videoconference. However, there was 1 technical difficulty in saving the video for 1 father, and thus, only audio was available for that visit. Detailed field notes were also documented for each visit by 2 staff members in attendance. The sample of caregivers who participated was mostly White (n=10, 83%) and married (n=8, 67%). They were equally dispersed amongst metropolitan, rural, and Appalachian areas (n=4 each, 33% each). Although most caregivers were biological parents (n=9, 75%), there were 2 grandfathers and 1 legal guardian/adoptive parent. At the time of the interview, young survivors of childhood cancer were between the ages of 3 and 9 years. Demographic characteristics are presented in Tables1  2.

**Table 1.** Demographic characteristics of caregivers who participated in usability testing.

Characteristics of caregivers	Women caregivers (N=7)	Men caregivers (N=5)
Caregiver age (years), mean (SD)	39.00 (4.04)	47.80 (5.93)
Number of children in the home, n (%)		
One child	2 (29)	2 (40)
Two or more children	5 (71)	3 (60)
Race, n (%)		
White	6 (86)	4 (80)
Black or African American	1 (14)	—
Prefer not to respond	—	1 (20)
Ethnicity, n (%)		
Hispanic or Latinx	1 (14)	—
Not Hispanic or Latinx	5 (72)	3 (60)
Prefer not to respond	1 (14)	2 (40)
Geographic area, n (%)		
Metropolitan	3 (42)	1 (20)
Rural	2 (29)	2 (40)
Appalachian	2 (29)	2 (40)
Educational attainment, n (%)		
High school	1 (14)	—
Some college	1 (14)	2 (40)
College	4 (57)	2 (40)
Graduate or professional	1 (14)	1 (20)
Annual family income, n (%)		
US $25,001-$50,000 per year	3 (42)	1 (20)
US $50,001-$75,000 per year	1 (14)	1 (20)
US $75,001-$100,000 per year	1 (14)	—
US $100,000 or more per year	2 (29)	2 (40)
Prefer not to respond	—	1 (20)
Relationship status, n (%)		
Single	—	1 (20)
Married	5 (71)	3 (60)
Divorced	1 (14)	—
Living with someone	1 (14)	1 (20)
Relationship to young survivor, n (%)		
Biological parent	6 (86)	3 (60)
Grandparent	—	2 (40)
Legal guardian	1 (14)	—

**Table 2.** Demographic characteristics of children from families (N=10) who participated in usability testing.

Characteristic	Value
Age at time of participation (years), mean (SD)	5.80 (1.69)
Age at diagnosis (years), mean (SD)	2.30 (1.55)
Cancer diagnosis, n (%)	
Brain tumor	2 (20)
Leukemia or lymphoma	3 (30)
Non-CNS^a^ solid tumor	5 (50)
Cancer treatment history, n (%)	
Chemotherapy	7 (70)
Radiation	1 (10)
Neurosurgery	2 (20)
Documented in the electronic medical record problem list, n (%)	
Speech difficulties	4 (40)
Gross motor difficulties	3 (30)
Fine motor difficulties	2 (20)
Neuropathy	4 (40)
Learning or attention-related difficulties	3 (30)
Emotional or behavioral challenges	7 (70)

a CNS: central nervous system.

### Preliminary Impressions

#### Passive Use Feedback

During the 5 minutes of unguided use of the platform, the top viewed pages included: “What changes to expect” (module 1 landing page, n=9), “telling my child” (n=6), “growth of my child” (n=5), and “effects of health condition” (n=5; refer to File S3 in Multimedia Appendix 1 for heatmap of engagement). Given that these were several of the first pages of the first module, it seemed that most caregivers preferred to review the content in chronological order. Notably, some caregivers explored the module overview page and selected topics based on title (n=4), while fewer reviewed the resources page (n=3), and only 1 caregiver watched the tutorial video. Caregivers noted positive initial impressions of the platform, including that it was user-friendly and liked the overall design. For example, one mother of a 7-year-old described: “Anyone with young children would probably want to go through it and play around.” Similarly, a grandfather of a 7-year-old reported: “I think this is a wonderful program, and would be very beneficial for families going through this with their child.” Caregivers also made suggestions for improvement, including greater accessibility (eg, captioning on video and audio versions of content) and tailoring of content for this population. For example, related to the integration of audio content, one mother of a 7-year-old described: “Some parents are just so tired, they don’t wanna sit there, and their eyes just look at a screen. They just want to sort of listen.”

#### Scenario-Based Feedback

Caregivers spent an average of 17.83 (SD 6.28; range 8‐31) minutes completing the scenario (module 1.2: How do I connect with my child?). They made several positive comments about the videos, design, and relatability of the content. For example, one mother described: “It’s easy to read, not too many words, which is nice, because I feel like when you wanna learn information…you wanna get to what’s important.” Caregivers also made suggestions for improvements, including the mobile navigation and ease of starting the modules.

### Summative Feedback

#### SUS Results

All but one mother, who used PLAY on a smartphone, found the platform easy to navigate and usable (92% SUS score>68, indicating acceptable usability; total mean 82.2). Both men and women caregivers reported similar levels of overall usability (men: mean 82.50, SD 6.85; 100% SUS score>68 and women: mean 83.21, SD 18.75; 86% SUS score>68).

#### Device Usage

Half of the caregivers used a laptop or desktop PC to access the platform (n=6, 50%), while 5 (42%) used a smartphone, and 1 used an iPad. Caregiver responses for their preferred device to access the platform mirrored their usage for the usability testing. However, all caregivers reported that they had access to a smartphone, while 11 (92%) reported having a laptop or desktop PC, and 9 reported having an iPad/tablet (75%). Usability scores were lower and more variable for caregivers who used a smartphone as compared to those who used a PC (smartphone user: mean 79.50, SD 20.95; PC user: mean 84.16, SD 8.76). However, these group differences were not statistically significant (*t*_9_=−0.50; *P*=.63), nor were the scores below the SUS threshold (<68) for acceptable usability.

#### Reaction Cards

**Figure 3.**
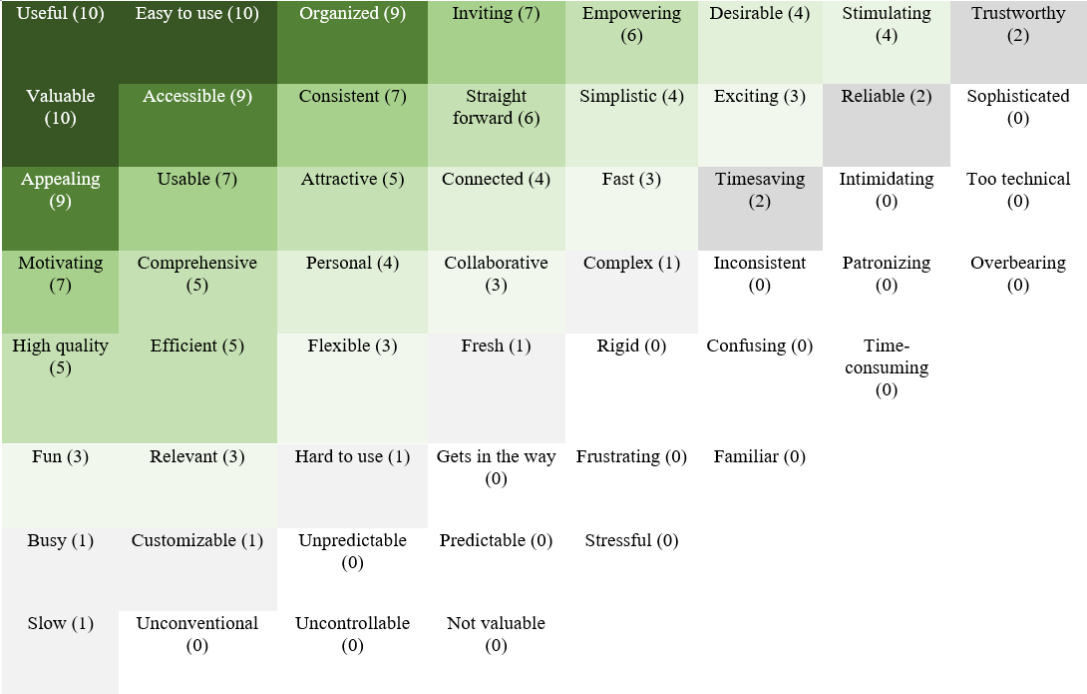
Heatmap of reaction cards following usability testing regarding the website. The numbers in parentheses indicate the number of participants who selected the adjective, with the most highly endorsed adjectives in the top right corner.

Caregivers used primarily positively valenced adjectives to describe their impressions of the platform, including useful, valuable, easy to use, accessible, and appealing. A heatmap displaying the frequency of adjectives selected is presented in Figure 3.

#### Rapid Analysis

The summative interview data yielded several themes, which are displayed in Table 3 and throughout the “Results” section.

**Table 3.** Rapid analysis themes on the innovation design based on Preparing for Life and Academics for Young Survivors program prototype.

Themes	Enablers	Overarching suggestions for improvement
Overall impressions	Easy-to-use interface Positive toward the platform Simple/straightforward design (M)^a^	Reduce/reformat text Changes to videos Message boards with caregivers Chat feature with professionals Other websites/resources to learn about specific tumor types and treatments Links to local support groups Directory for resources Discounts for parents to destress (eg, spa/massages) Link to electronic medical record
Perceived usefulness	Found to be useful for supporting and teaching responsive parenting skills	Add more realistic scenarios dealing with stress Use “coach” vs “therapist” Add more real-life examples of dealing with stress as a parent List of questions to ask at follow-up appointments Ways to destress before scans/follow-up appointments More information about feeling misunderstood by friends Improvements to videos, including a greater variety of ages, closed captioning, and video testimonials about the program
Design and layout	Favorable design	Reduce text: more images and graphic representation of concepts Visual progress bar Bold key points More accessible “take-away points” More concise instructions on exercises More layman’s terms Option to have text read aloud
Navigation	Easy-to-use interface	Improved mobile menu and mobile accessibility Linkages between modules
Preferred format and timing	Internet-based and easily accessible format	Offer a platform to women caregivers shortly after diagnosis (W)^b^ Offer a platform to men caregivers on the transition off treatment (M)

a M indicates theme for men caregivers only.

b W indicates theme for women caregivers only.

#### Overall Impressions

Overall, most caregivers noted that they found the platform “easy to use,” including comments that the platform was “simple,” “straightforward,” and “user-friendly.” A few caregivers made suggestions about “reducing and reformatting text” to make it easier to digest, such as including more comics and images to “break up the text,” bolding key points, and including key takeaways that are easily accessible for future reference in bullet point format. Specific enablers and suggestions for improvement are presented in Table 3. Caregivers also recommended “improvements to the videos,*”* such as closed captioning and showing a greater variety of age groups and developmental periods. One mother of a 7-year-old survivor noted that she would prefer if the videos were “more fun” and “less forced.” Caregivers “denied concerns about privacy*”* while using the platform, with a few noting that it seemed “trustworthy” with the ability for an individual log in.

#### Perceived Usefulness

Most caregivers described that the platform “could be useful*.*” One father of a 3-year-old survivor described that he liked that the platform showed: “the various challenges that you’re about to embark upon and puts them into nice boxes...modules that are easily digestible.” Several caregivers noted that some of the content was familiar to them. On the other hand, caregivers described that they may benefit from learning how to “be more specific with praise” in interacting with their children. One grandfather of an 8-year-old survivor noted that presenting this information in this online format reduces defensiveness, as compared to having someone else tell you what to do. Specifically, he described:

*I think it’s (platform) better than hearing their parents tell them how to do it or having another person tell them how to do it directly...because then they…kind of have some more time to process what they’re seeing and viewing...I think that it’s good because it gives them an opportunity to process versus having just people saying it to their face and then being defensive. With a module like this, they can…be defensive to the module, but nothing is going to talk back to them.*

[Grandfather of an 8-year-old survivor]

One suggestion for improvement included more “real life examples” of caregivers managing stress in the moment*,* such as taking a break or a walk. One mother of an 8-year-old survivor suggested that it would be important to incorporate relatable examples:

*Real-life scenarios, real-life practice, and examples that we (caregivers) can do with our child that are just kind of practical, like even if it’s not traditional. Just a reminder that when you go out to a park with your child today, why not ask them about the colors they see or count how many swings are at the swing set? Just a reminder of simple things we (caregivers) can do.*

[Mother of an 8-year-old survivor]

Caregivers also described that the platform may be useful for *“*enhancing teachers’ understanding*”* of cancer, as well as caregivers’ understanding of late effects of cancer treatment and the impact of cancer on siblings. They also noted that the platform would, in the words of a mother of a 6-year-old survivor, help caregivers be “more empowered to be advocates for their child.”

#### Design and Layout

In relation to the design and navigation, most caregivers commented “positively,” describing the platform as “pleasant,” “relatable,” and saying that the “preschool” designs are fitting for the age group.

#### Navigation

Consistent with the reduced SUS scores for the mobile version, a few caregivers suggested improvements for mobile usability, including expanding the mobile menu and navigating to start the modules.

#### Preferred Format

Most caregivers (9/12) described that individual coaching in a future intervention program “could be helpful” and should be offered as “an option” for families to provide “another level of support.” Still, one grandfather of a 5-year-old survivor noted that it is important that caregivers do not feel “graded” or “judged.” Caregivers also emphasized the need for ease of scheduling these appointments, such as evenings, weekends, and flexibility with appointments (eg, last-minute cancellations).

With respect to offering text messaging as a supplement to the internet-based modules, caregivers described that they could be helpful as reminders of skills learned on the platform. Caregivers noted that it would be important to ask about preferences for frequency (eg, weekly and monthly) to prevent these reminders from becoming overwhelming.

#### Preferred Timing

Regarding the timing of a responsive parenting program, caregivers expressed a variety of opinions, which differed by the gender of the caregiver. Most women caregivers described desiring this type of support within a few months of diagnosis. One mother of an 8-year-old survivor described:

*Definitely not the first month…we were in the hospital for two weeks. It was a whirlwind. Parents were taking time off work. The family’s coming to town. No one knows what’s going on...and then you’re sort of in denial for the first month, and you’re in fight or flight mode. So, I would say probably month two or three would be a good time, because now you’re sort of learning, learning how your kid responds to some of the treatment.*

[Mother of an 8-year-old survivor]

Another mother of a 6-year-old survivor commented about feeling somewhat helpless at the beginning and that there is “not much action you can take right away, except for learning how to help your child.” In contrast, most men caregivers reported that they would prefer to receive this support after the conclusion of treatment. For instance, one grandfather of a 5-year-old survivor described the intensity of the acute treatment period as: “It’s like you’re hit with a sledgehammer.”

## Discussion

### Principal Findings

This mixed methods study involved initial usability testing of a high-fidelity prototype of an internet-based responsive parenting intervention program called PLAY, designed to support caregivers of young survivors of childhood cancer. Overall, all but one caregiver found PLAY usable (92% reported SUS scores ≥68). Acknowledging the limitations of the SUS as a summative measure, we supplemented this analysis with qualitative feedback and the use of reaction cards [42,43]. Caregivers primarily selected positively balanced adjectives (eg, useful, valuable, easy to use, accessible, and appealing) to describe the program. These results are promising and comparable to other internet-based support and educational resources for caregivers of children with various chronic and developmental conditions and for those from rural and remote areas (eg, autism and asthma) [44-48]. Similarly, in the previous pilot of the 6-session intervention with caregivers of young children with TBI, all 11 families who completed the full 6-session intervention reported high satisfaction with the program [27].

This study, strengthened by a mixed methods approach to usability testing, yielded valuable and detailed information related to the innovation design (ie, CFIR) [24] of PLAY. Caregivers identified key aspects to tailor the program for the unique needs of the young survivors of childhood cancer, such as resources specific to cancer and late effects, examples and resources related to advocacy for their child in health care and school settings, and the importance of the caregiver managing their own stress and uncertainty (eg, “scanxiety”) in their capacity to implement responsive parenting skills. Other general suggestions included increasing usability, offering coaching and texting as optional supports, and considering intervention timing, which differed by gender of the caregiver. With respect to usability, caregivers repeatedly underscored the necessity of PLAY being highly mobile-friendly, practical, and brief, given multiple competing demands on caregivers’ time and attention. Most caregivers agreed that the length of the module was appropriate (approximately 15‐20 minutes), and all caregivers reported having access to a smartphone and a preference for mobile usability. This is comparable to other studies with caregivers of young children with autism, in which mobile usability was a top priority [45]. The Pew Center also highlights that most adults living in both metropolitan (89%) and rural (80%) areas own a smartphone [49]. Still, both quantitative (ie, SUS) and qualitative data indicated that improvements were needed in the mobile usability and design of the platform, such as easier navigation to the main menu on the mobile version. Other suggestions to improve accessibility included closed captioning on videos and options for the text to be read aloud on each module page. These suggestions are in line with similar studies of digital health interventions (DHI) for caregivers [50,51]. Furthermore, these adaptations would assist in bridging the “disability digital divide” [52].

Most caregivers described receptiveness to coaching from a trained professional and occasional text message reminder, in addition to the self-guided platform resource. Caregivers emphasized that these supports should be optional and individually tailored in terms of frequency. They also recommended that coaching should be flexibly scheduled to increase accessibility for busy families with young children (ie, evenings and weekends). In studies of telehealth interventions for caregivers of children with autism [46], caregiver retention and positive outcomes were superior following weekly contact with a therapist, as compared to self-guided. Multimodal interventions may be especially needed, given the documented decline in caregiver participation over the course of internet-based positive parenting programs [53]. One consideration for future intervention development is that videoconference coaching may require high-speed broadband coverage, which may reduce accessibility for this benefit for rural families, as approximately 73% of rural households have home subscriptions [49].

Unexpectedly, there were some gender differences in the preferred modality and timing of this type of responsive parenting resource. Specifically, women caregivers preferred resources in the immediate period after diagnosis, while men caregivers preferred resources after cancer treatment had concluded. Although not universal even amongst our participants, this may be attributed to the common division of responsibility and typical gender roles after a child is diagnosed with cancer. Mothers often directly support the child in the hospital, while fathers and grandfathers often maintain their work schedule and household responsibilities [54,55]. Fathers may prefer to wait until after treatment is concluded when they feel more involved and included in care. Furthermore, there may be gender differences in how caregivers cope and adjust to the diagnosis and treatment [56], which may affect readiness for intervention. Still, this difference in preferred timing of resources may also be attributed to the difference in mean age and caregiver type, such that there were two grandfathers but no grandmothers in our small sample. At least one grandfather reported that he preferred to receive information about responsive parenting through the self-guided platform as opposed to coaching due to feeling defensive or “judged.” This aligns with previous work suggesting that fathers and other caregivers who are men may face unique barriers in help-seeking [57] and are often excluded from parenting intervention research [58]. Given that preferences in timing and modality may differ across family members, DHI such as PLAY might be offered to families earlier and more periodically throughout the illness trajectory, with careful framing of the parenting intervention to encourage father engagement.

### Limitations

Although this usability testing study offers several insights for the refinement of digital health platforms for caregivers of young children with chronic health conditions, several limitations should be considered. First, although we attempted to conduct stratified sampling, our post hoc testing indicated possible ascertainment bias with our subsample, including slightly older caregivers, who may have differing usability and information preferences than younger caregivers. Similarly, our recruitment rate of approximately 40% was relatively low, yet higher than a similar study with caregivers of young children following TBI (25%) [27]. This highlights the inherent challenges of engaging with caregivers of young children with complex medical needs, requiring future work to identify additional ways to engage and provide accessible psychosocial care to this underserved and sometimes hard-to-reach group. Second, although our sample size was sufficient to assess usability experience [39], we were not powered to examine subgroup differences based on type of caregiver or diagnostic group. Third, we used a rapid analysis approach [34], in which we were unable to calculate interrater reliability between coders. The interviewers and coders were part of the study team and, thus, their probing during interviews and observations in rapid analysis may have been biased. Still, the usability was assessed with multiple methods (ie, standardized measures, reaction cards, and “think aloud” procedures), in line with current research recommendations [43]. Finally, the single-session usability testing limited the review of only one module in detail (ie, responsive parenting). Future work will need to obtain perspectives of the PLAY intervention comprehensively (ie, other modules listed in File S3 in Multimedia Appendix 1).

### Future Study Plans

Overall, internet-based responsive parenting interventions to support caregivers of young children may have promising transdiagnostic applications. However, creative problem-solving may be needed to improve future engagement, uptake, and implementation. Guided by the CFIR framework and designing with implementation in mind, our next step (ie, phase 3) will involve an iterative co-design process [59] with a CAB (n=13) and a multidisciplinary Healthcare Provider Advisory Board (HPAB; n=11). Co-design involves close collaboration between researchers, designers, and users, who serve as the experts due to their experiences [59]. For our proposed study, the research study team will partner with both the CAB and HPAB during monthly meetings held via videoconference to review and discuss feedback on each module of the PLAY program to allow for in-depth feedback and tailoring of each page of the intervention. For example, the self-care and relationships (refer to File S3 in Multimedia Appendix 1) modules focus on how the caregiver and their relationships have been affected, as well as strategies that may help (eg, relaxation strategies, and “I statements”). Both advisory boards will collaborate with the study team to develop and iteratively refine the graphics, videos, exercises, and text-based content to reflect their lived experiences and clinical expertise. Additional single-session user-experience testing with another group of caregivers will be conducted to ensure adequate usability (SUS ≥68) with the refined and tailored PLAY program. A pilot of the PLAY intervention will test feasibility and acceptability with caregivers of young survivors of childhood cancer, with a small randomized controlled trial to follow. A future larger randomized controlled trial will determine whether the program improves responsive parenting behaviors over several months and leads to downstream effects on neurodevelopmental outcomes for young survivors of childhood cancer. Future work will also identify barriers and facilitators in the inner and outer settings from the CFIR framework to support the adoption of the PLAY intervention in clinical practice.

### Conclusion

Designing equitable digital health interventions demands the active and ongoing engagement of individuals with lived experience across all stages of design, particularly ensuring the inclusion of underrepresented groups to optimize success.

## Supplementary material

10.2196/70055 Multimedia Appendix 1 High-fidelity prototype of the Preparing for Life and Academics for Young Survivors (PLAY) program module 1.2 used during usability testing, semistructured interviewing guide, and heatmap of pages accessed during passive usability.

## Abbreviations:

CAB: Caregiver Advisory Board
CFIR: Consolidated Framework for Implementation Research
DHI: digital health interventions
GROW: Gaining Real Life Skills Over the Web
HPAB: Healthcare Provider Advisory Board
PALS: Play and Learning Strategies
PLAY: Preparing for Life and Academics for Young Survivors
REDCap: Research Electronic Data Capture
SUS: System Usability Scale
TBI: traumatic brain injury
