# Supplementary material for: Usability Testing of an Internet-Based Responsive Parenting Program for Caregivers of Young Survivors of Childhood Cancer Living in Rural and Appalachian Communities: Mixed Methods Study
Source: JMIR Pediatr Parent. 2025 Aug 5;8:e70055. doi: 10.2196/70055 (PMC12516296; doi:10.2196/70055)
Supplement: Multimedia Appendix 1 [file pediatrics-v8-e70055-s001.docx]

**File S1**: High-fidelity prototype of Preparing for Life and Academics for Young Survivor (PLAY) module 1.2 that was used during usability testing.


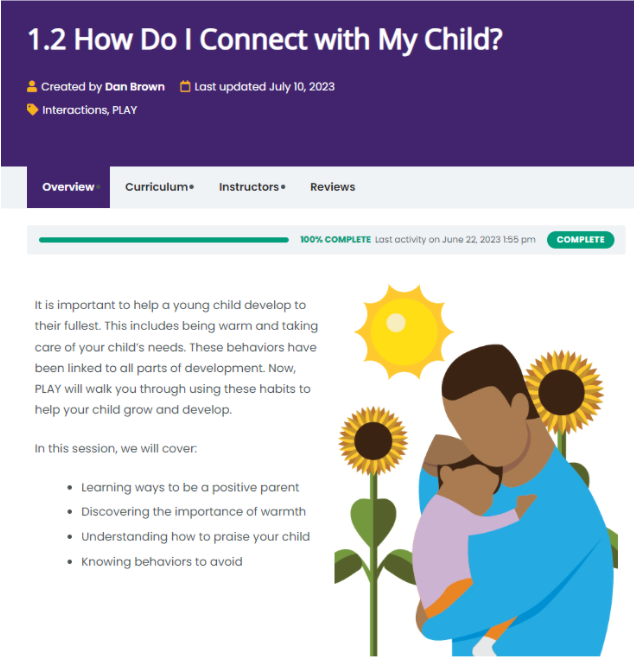


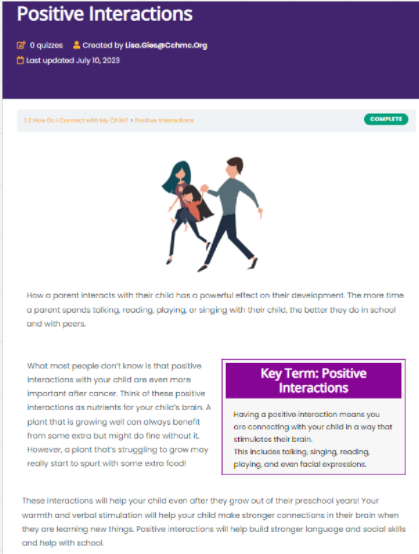


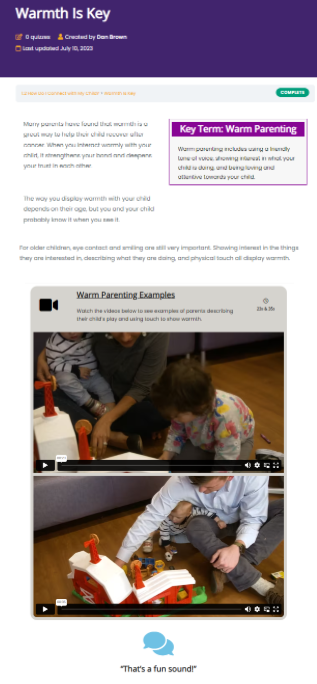


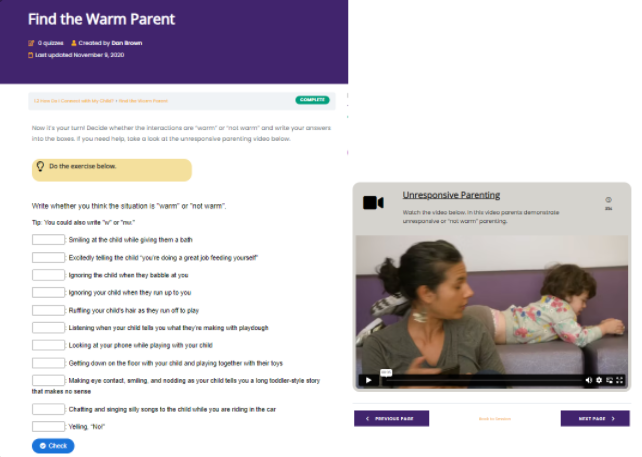


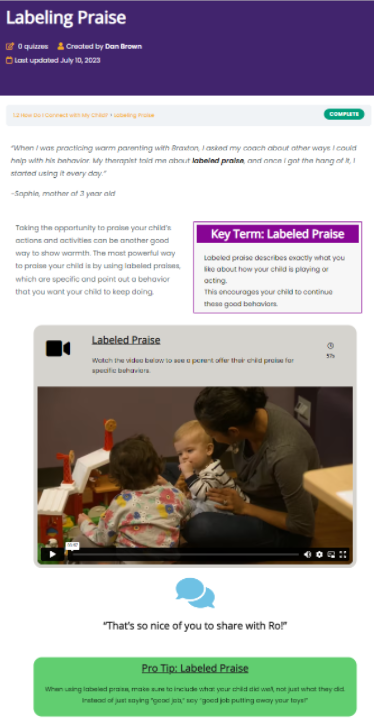


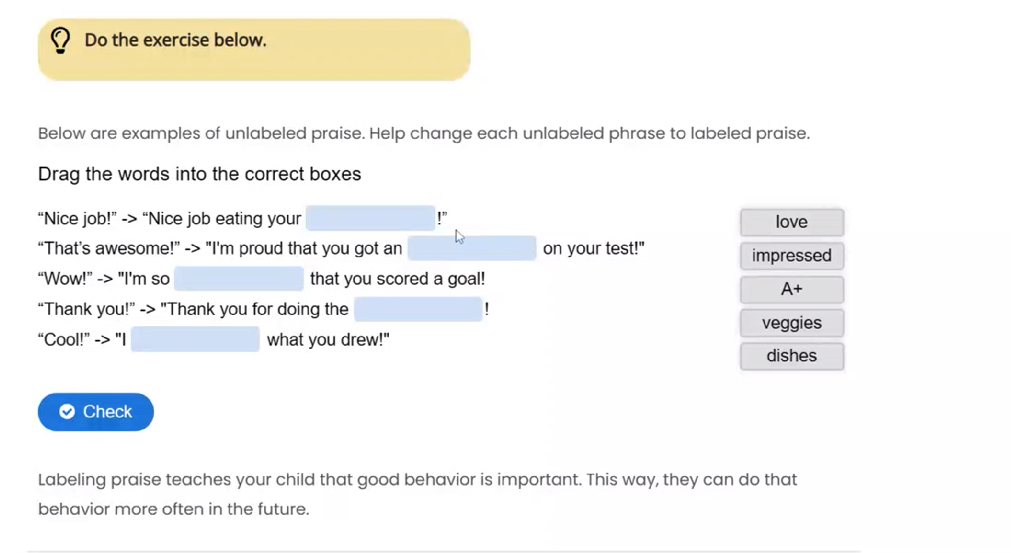

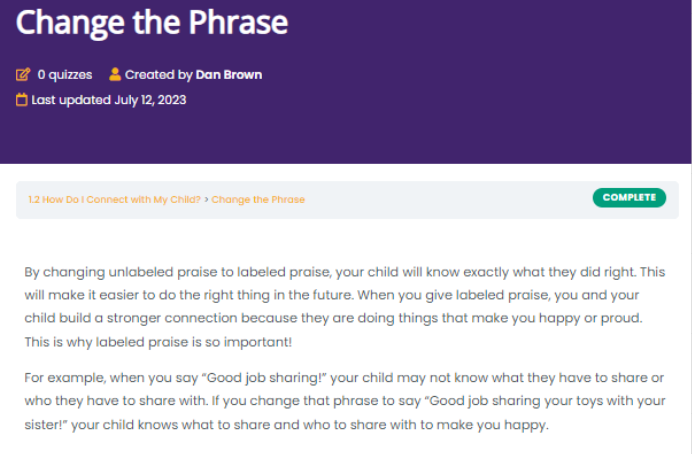


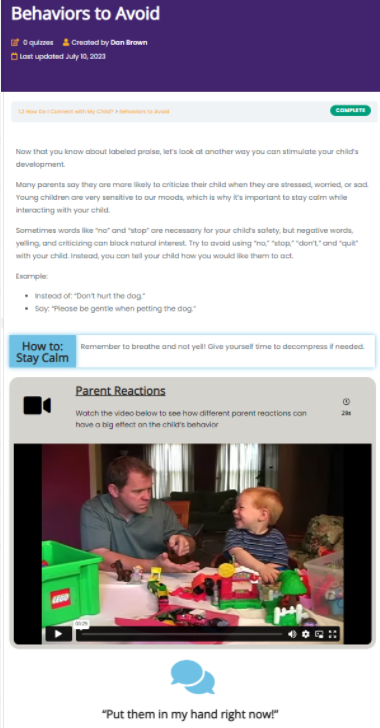


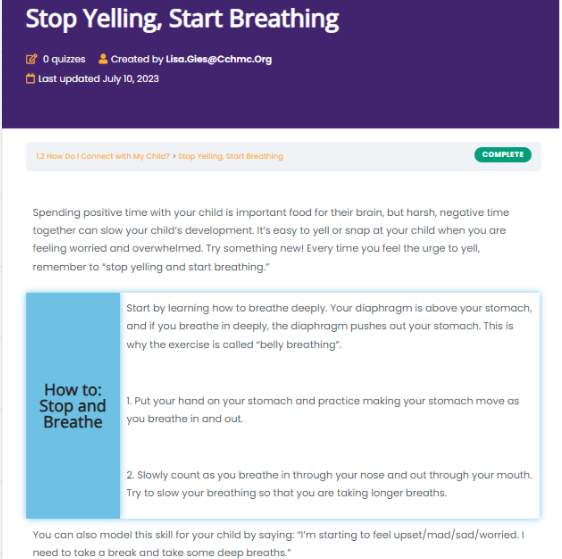


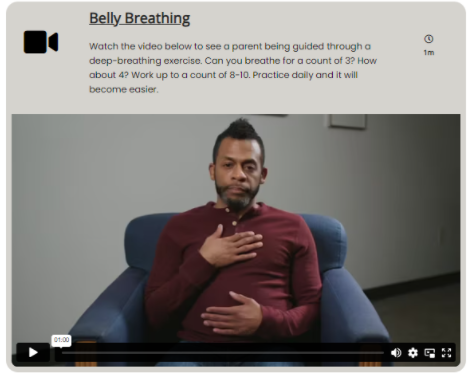


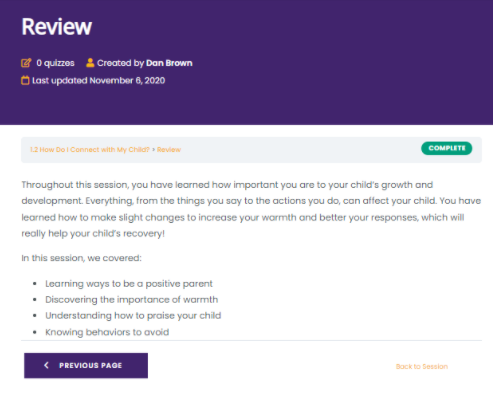


**File S2:** Semistructured interviewing guide.

1. What do you think of the GROW program website? (Probes: What would you change? How could it be improved?)
2. How (if at all) do you think the website could be useful in supporting parents of young children with learning positive parenting skills? (Probes: How could this be useful/not useful for parents of young children with cancer? What about helping parents get their young child ready for school? What about with a parent’s own stress?) How do you think information could be used for other members of the healthcare team or teachers? [**Perceived Usefulness (TAM)]**
3. What do you think about the overall design/how it looks? (Probes: Favorite aspect/least favorite aspect?) [**Design and layout]**
4. How easy was it to get around the website? How natural is the website to use? (probes: do you think the website is easy or hard to use compared to the other websites you use? What troubles did you have while learning to use the website? Do you think the website was hard to use? Did you get “stuck” on any parts of the website? Did you become confused at any point using the website? If so, can you explain? [**Perceived Ease of Use** (TAM) / **Difficulty of use (Burden scale) / Ease of Use (Usability)]**
5. Did you have any concerns about privacy while using the website? (Probes: such as information stored in external server/website?). [**Privacy (Burden scale)**
6. What have you learned about positive interactions from the website? (Probes: How can parents praise their child? What should parents avoid?)
7. What are your initial impressions/thoughts about parent coaching for families of children with cancer? (Probes: What questions do you have about this type of program? Would this be useful? Would this have been useful for your family? What aspects would be useful/not useful? Parent/child play time, reading strategies, teaching ways to guide child’s learning, and teaching how to manage own stress? Other aspects that parents need help with?)
8. What would work/would not work about this program for parents of children with cancer? (Probes: What about the format? Length? Time since diagnosis/age of child?)

*A final aspect of the intervention might include text messaging. We are now going to send you an example text message in the chat of what a parent participating in the program would receive:*

GROW tip of the day: Try using specific, labeled praises while playing with your child, such as “Great job waiting your turn!”

1. What would you think if you received something like this? (Probes: Would you prefer a text message or email? Would this be helpful or not helpful in reminding you of skills learned on the website?)
2. Of all the things we discussed, what is the most important to you?
3. What have we missed? Anything you would like to add?

**Note: Constructs bolded and in parentheses refer to the Technology Acceptance Model**

**File 3:** Heatmap of pages accessed during passive usability.


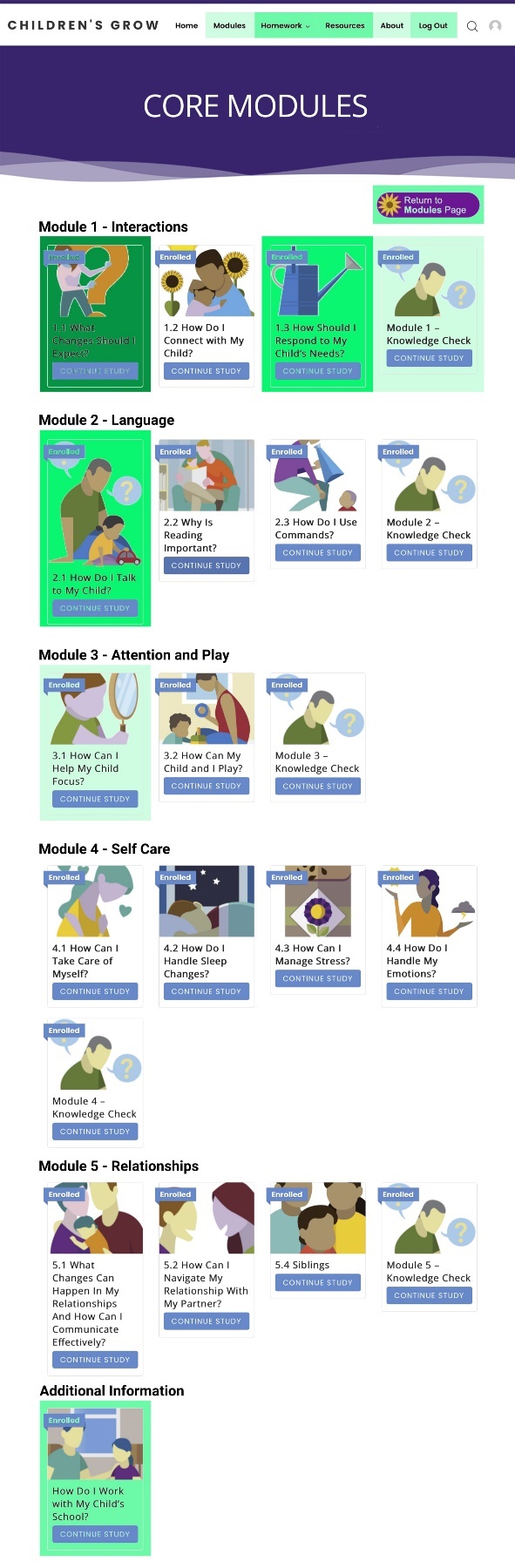


Note: Darker green highlight indicates more frequent clicks during unguided use
